# Decoding the Factors Influencing Parental Vaccine Decision-Making: Insights from Saudi Arabia on COVID-19 Vaccination for Children—Lessons for Future Pandemics

**DOI:** 10.3390/vaccines14090745

**Published:** 2026-08-27

**Authors:** Lamyaa Kassem, Mohammed Saif Anaam, Fatimah A. Aldaiji, Fatimah A. Alqarzaee, Farah A. Almogarri, Hana A. Almansour, Nouf A. Almutairi, Mohamed Hassan Elnaem, Hadiah Almutairi, Sulaiman Ibrahim Alsohaim, Khalid Siddeeg, Waleed M. Altowayan

**Affiliations:** 1 Department of Pharmacy Practice, College of Pharmacy, Qassim University, Buraydah 52571, Qassim, Saudi Arabiak.mohammad@qu.edu.sa (K.S.); 2 United Pharmaceuticals Company, Jeddah 21411, Makkah, Saudi Arabia; 3 School of Pharmacy and Pharmaceutical Sciences, Faculty of Life and Health Sciences, Ulster University, Coleraine BT52 1SA, UK

**Keywords:** vaccine reluctancy, COVID-19 vaccines, cross-sectional study, questionnaire, Saudi Arabia

## Abstract

**Background**: In Gulf nations such as Saudi Arabia, where COVID-19 vaccination is optional, parental decision-making regarding vaccinating children is critical. Understanding the psychological factors that drive these decisions can shape future vaccination strategies. **Methods**: Between November 2021 and January 2022, a cross-sectional, web-based validated survey was conducted using chain-referral (snowball) sampling across multiple social media platforms. A total of 2176 parents residing in Saudi Arabia with children aged 5–18 years participated. The 5C psychological antecedents (confidence, complacency, constraints, calculation, and collective responsibility) were assessed. Multinomial logistic regression was employed to determine factors influencing parental decision-making across three child age groups: 5–11 years only, 12–18 years only, and both age ranges (5–18 years). **Results**: Overall, 77.9% of parents agreed to receive all scheduled doses of the COVID-19 vaccine themselves, while 58.6% agreed to vaccinate children aged 12 years or older, and only 41.9% agreed to vaccinate children younger than 12 years. Fathers were significantly more likely to accept vaccination [Odds ratio (OR) = 17.48, *p* = 0.01] compared to mothers, who showed higher reluctance (OR = 15.08, *p* = 0.01). Having a child aged 5–11 years increased reluctance threefold (OR = 3.16, *p* = 0.01). Parents who believed that returning to school was safe showed increased vaccine acceptance (OR = 4.32, *p* = 0.01). Confidence and collective responsibility were the strongest positive predictors of vaccine acceptance, particularly for younger children, with OR estimates of 1.23–1.53 for confidence and 1.33–2.13 for collective responsibility. Conversely, extensive information seeking (calculation) was associated with lower acceptance (OR = 0.09, *p* = 0.03). A history of COVID-19 hospitalization among family or friends was significantly associated with increased parental reluctance (*p* = 0.03). **Conclusions**: This study identifies key psychological and demographic factors influencing parental decisions regarding COVID-19 vaccination for children. Building parental confidence and fostering collective responsibility are essential for increasing vaccination rates, particularly among parents of younger children aged 5–11 years. Healthcare authorities should address vaccine safety concerns, provide clear and accurate information, and tailor strategies to parents who remain hesitant.

## 1. Introduction

Due to the COVID-19 pandemic, the world economy, healthcare institutions, and daily life have faced tremendous disruptions. Vaccination has emerged as a critical strategy for curbing viral spread and minimizing its devastating consequences. Kingdom of Saudi Arabia (KSA), like many other nations, has prioritized COVID-19 prevention and public health protection. The race to produce COVID-19 vaccines necessitated rigorous evaluations by regulatory agencies such as the FDA, EMA, and Gulf States, leading to the licensure of numerous adult vaccines. Subsequently, immunizations were permitted for adolescents aged 12 to 18 years, and later, half-dose vaccines for young children aged 5 to 11 years were authorized [1,2]. On 14 March 2023, underscoring the continued expansion of vaccination efforts to younger age groups, the FDA approved the Pfizer-BioNTech vaccine for infants and toddlers aged 6 months to 4 years [3,4].

Several factors affect COVID-19 vaccination rates, including individual risk–benefit evaluations and motivations to prevent pandemic spread or safeguard family members [5,6]. The majority of parents actively weigh the risks and benefits of immunizing their offspring, with many expressing concern about the COVID-19 virus and viewing vaccination as essential for ensuring their children’s health. Consequently, parents’ perspectives on vaccinating their offspring must be determined, and their decision-making variables must be studied in depth [7].

Complex situational factors, including scientists’ pandemic-prevention efforts, further complicate this decision-making process. Additionally, media and social media platforms can misinform parents [8]. The political and societal effects of the pandemic increase the urgency of regulating and ending the global health crisis. Complete economic recovery depends on the safe return of all children to school and the capacity of parents to resume their regular work schedules without compromising their children’s safety [9,10].

The dynamics of COVID-19, characterized by heightened transmissibility and uncertain long-term effects, have introduced new considerations and complexities into parental decision-making regarding vaccination. By analyzing the various psychological elements impacting parental willingness to vaccinate their children, tailored strategies can be developed to resolve concerns and promote vaccination acceptance. These insights are essential for tackling both present and future vaccine-related challenges and will contribute to ending the pandemic, enhancing public health, and supporting economic restoration [11].

As the first distribution of COVID-19 vaccines was authorized under pandemic emergency rules, individuals and parents faced additional pressure to make rapid vaccination decisions. Some parents may hesitate due to safety and efficacy concerns; however, it is important to note that emergency use authorization involves rigorous testing and evaluation. Public health officials and healthcare providers play a crucial role in providing accurate information and support to parents making informed decisions about vaccinating their children against COVID-19 [1,9,10,11]. Understanding these factors is key to developing strategies to combat vaccine reluctance and ensure individual and community safety [11].

Fortunately, researchers have developed robust techniques to understand the complex psychological variables underlying vaccine reluctance. The 5C scale has become a widely accepted instrument for evaluating the psychological drivers underlying vaccine refusal and for interpreting self-reported vaccination behaviors at the population level. The 5C scale helps elucidate psychological factors driving vaccine reluctance and supports the development of evidence-based measures to overcome it [12,13]. The 5C scale was recently translated and validated in Arabic, making it a versatile and useful tool for understanding the psychological dynamics of vaccine reluctance in diverse cultural contexts [14,15]. The 5C scale evaluates five key factors: Complacency (low perception of disease risk), Constraints (physical and psychological barriers making vaccination inconvenient), Calculation (active searching for vaccine information), Collective Responsibility (willingness to receive vaccination to protect others), and Confidence (trust in vaccine safety, efficacy, and healthcare providers) [16].

This study aims to evaluate the incidence of parental vaccine reluctance, examine differences in attitudes toward COVID-19 immunizations among parents of children across varying age groups, and identify the factors influencing parental decisions regarding COVID-19 vaccination for their children. The findings will provide useful insights for future vaccination implementation strategies and pandemic preparedness.

## 2. Materials and Methods

### 2.1. Study Design

A cross-sectional, web-based survey was conducted between November 2021 and January 2022 to investigate the determinants of parental decisions regarding COVID-19 vaccination for children in Saudi Arabia. A chain-referral sampling technique (commonly known as snowball sampling) was employed to recruit participants through multiple social media platforms, including Facebook, Twitter, LinkedIn, and WhatsApp. Sample size calculation was dependent on the expected level of parental vaccine hesitancy in KSA, which was estimated at 20% in a previous study [17]. Using an online sample size calculator designed for cross-sectional studies [18], a minimum required sample of 1448 participants was identified. To account for potential clustering effects and enhance statistical precision, this calculated figure was multiplied by a factor of 1.5, yielding a target sample size of 2176 participants.

Eligibility criteria included Saudi Arabian parents whose children were aged between 5 and 18 years and had received at least one dose of a COVID-19 vaccine. Following data collection, 28 respondents were excluded because they either did not reside in Saudi Arabia or had children outside the specified age range. The remaining participants proceeded to complete the full survey.

### 2.2. Data Collection Tool

We developed a structured questionnaire based on previously validated tools [15,19]. After removing irrelevant items, we added new questions designed to capture parental motivation specifically related to protecting children from COVID-19.

At the beginning of the online survey, a welcome page provided a brief explanation of the study objectives, assured participants of the confidentiality of their responses, and clarified that proceeding with the survey would imply voluntary consent to participate. Respondents were informed that their continuation would authorize the use of their answers for research and publication purposes. No separate signed consent form was required, as participation was entirely voluntary and anonymous.

The final survey comprised 32 mandatory multiple-choice questions organized into three sections:

- Questions 1–12: Socioeconomic characteristics of participants.
- Questions 13–21: Participants’ personal experiences with COVID-19 disease and vaccination.
- Questions 22–32: Assessment of the five psychological antecedents of vaccination (the 5Cs). These constructs were evaluated using a Likert-style scale, with two to three questions dedicated to each domain.

Both Arabic and English versions of the questionnaire were validated by experts in pharmacy practice. The reliability of the instrument was assessed using Cronbach’s alpha coefficients. The overall Cronbach’s alpha for all items across all domains was 0.76, indicating acceptable internal consistency. Subscale-specific reliabilities were as follows: Confidence (α = 0.82), Complacency (α = 0.71), Constraints (α = 0.69), Calculation (α = 0.74), and Collective Responsibility (α = 0.85). These values fall within the recommended range (≥0.70) [20].

### 2.3. Statistical Analysis

All statistical analyses were performed using R software version 4.6.0 and IBM SPSS Statistics version 25.0 for Windows. Associations between categorical variables were examined using the chi-squared test. Participants were classified into three distinct groups based on their vaccination decision status: yes (willing to vaccinate), uncertain (ambivalent), and no (unwilling to vaccinate). This classification enabled identification of which of the 5C psychological constructs most strongly influenced decision-making. For analytical purposes, the “uncertain” and “no” responses were combined into a single “reluctants” group, while “yes” was labeled “acceptants”.

Multiple logistic regression was employed to determine the factors influencing parental decision-making across the outcome categories (yes (acceptants), uncertainty/no (reluctants). A threshold of *p* < 0.05 was established as the criterion for statistical significance. Parents were further stratified into three subgroups according to the age range of their children: children aged 5–11 years only, children aged 12–18 years only, and children spanning both age ranges (5–11 and 12–18 years). This age-based stratification followed the official World Health Organization (WHO) classification used during the successive approval rounds of COVID-19 vaccines for pediatric populations. To assess potential multicollinearity among predictor variables entered simultaneously into the regression models, we calculated the Variance Inflation Factor (VIF) for each predictor. All VIF values were below 5, with the majority ranging between 1.2 and 3.5, indicating no substantial multicollinearity concerns.

## 3. Results

### 3.1. Parental Sociodemographic Characteristics

**Figure 1:**
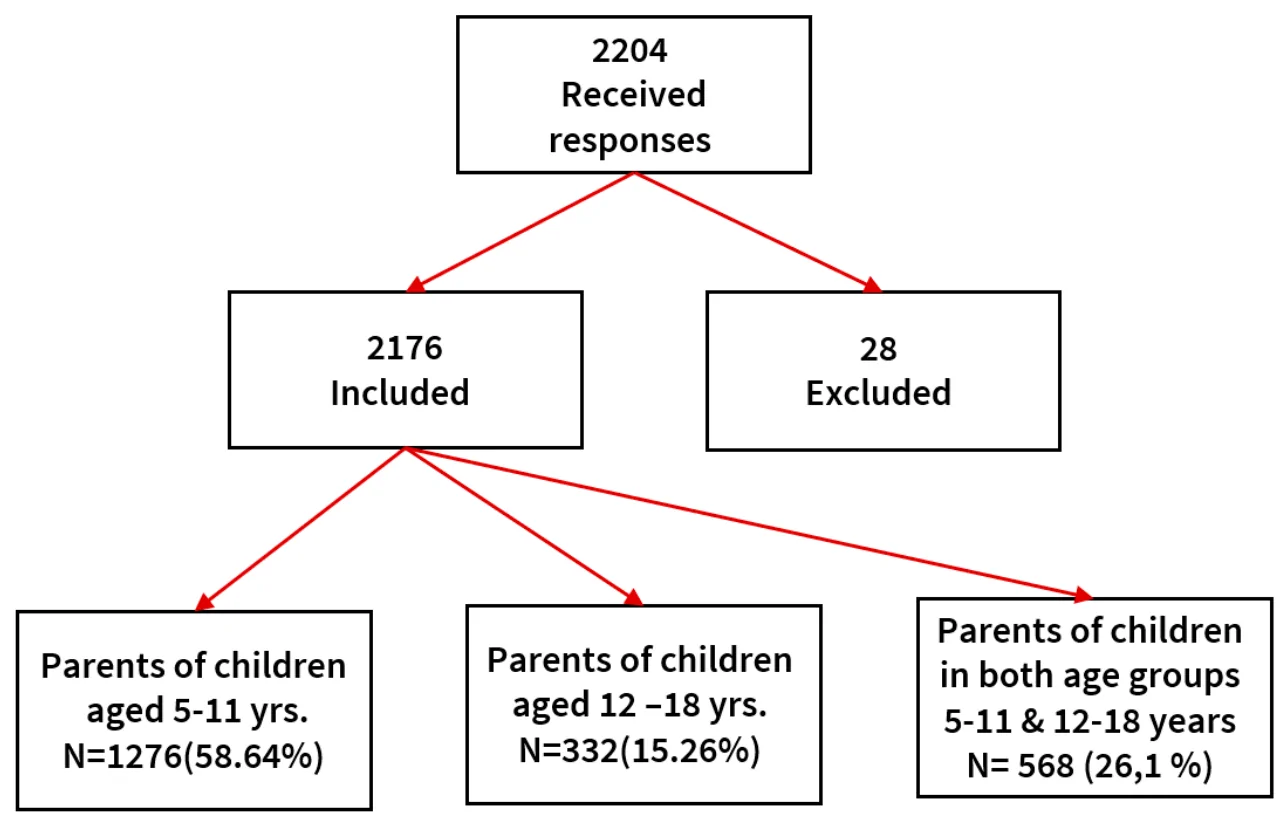
Participant stratification according to their children’s age range.

A total of 2176 parents were included in the final analysis and subsequently categorized into three groups according to their COVID-19 vaccination status (Figure 1).

The baseline demographic characteristics of the study population are presented in Table 1. All participants were residents of the KSA. The mean age of the sample was 38 years (standard deviation ± 12.4 years). The majority of respondents were non-healthcare professionals (n = 2036, 93.6%), Saudi females (n = 1384, 63.6%), and holders of a bachelor’s degree (n = 1272, 58.5%). Geographically, the largest proportion of participants resided in the Qassim region (n = 1236, 56.8%), followed by the Riyadh region (n = 464, 21.3%). The Qassim region, located in central Saudi Arabia, is primarily agricultural and known for its date palm cultivation and conservative social environment. Riyadh, the capital city, is the country’s largest urban center, characterized by its cosmopolitan population, diverse economy, and advanced healthcare infrastructure. All subjects had received the first dose of a COVID-19 vaccine, and about 92% had completed the second dose, reflecting a high level of vaccine acceptance and compliance with national immunization guidelines in the Kingdom.

### 3.2. Factors Contributing to Parental Reluctance to Vaccinate Children Against COVID-19

About half of participants (n = 1040, 47.8%) reported no history of COVID-19-related hospitalization for themselves or their family members. In contrast, 1212 respondents (55.7% of the sample) indicated that a relative or friend had died due to COVID-19 infection. A small proportion of participants (n = 280, 12.9%) reported that they or their children had a history of serious or chronic illness, which contributed to fear regarding receipt of the COVID-19 vaccine.

Two health-related factors were significantly associated with parental reluctance. First, a history of hospitalization due to COVID-19 infection, whether in the parents themselves, family members, or friends, was significantly associated with increased reluctance (*p* = 0.03). Second, exposure to news and debates on social media regarding the positive and negative effects of COVID-19 vaccines on human health also significantly influenced parental reluctance. No other health-related factors examined reached statistical significance.

### 3.3. Parental Intentions to Vaccinate Children and Perceptions Toward COVID-19 Vaccine

The study examined parental attitudes and intentions regarding COVID-19 vaccination for children. Overall, 65.9% expressed willingness to follow the Saudi Ministry of Health’s (SMOH) immunization schedule (*p* = 0.02). Intentions varied by age: 58.6% of parents planned to vaccinate children aged 12–18 years, while among those with children aged 5–11 years, 41.9% intended to do so, 20.2% had already vaccinated, and 29.2% of parents chose not to vaccinate their offspring (*p* = 0.001). Notably, 91.2% remained committed to routine SMOH vaccinations, though this was not significant (*p* = 0.16). A minority (14.9%) received anti-vaccination advice from healthcare providers. Over half (52.2%) believed vaccination was necessary for safe school return (*p* = 0.01). Child age and the perception that vaccination facilitates school attendance were significant determinants of parental intent. Positive responses were classified as “Yes”; negative or uncertain responses were grouped as “No/unsure.”

### 3.4. Parental Intentions to Vaccinate Children Based on Child Age Group

Parental intention to vaccinate varied substantially according to the age group of their children. Having a child aged 5–11 years exerted a significant effect on parental reluctance (i.e., “No or Unsure” response), with an OR of 3.16 (95% confidence interval [CI]: 1.57–6.37, *p* = 0.001) compared to those who answered “Yes” to vaccination intention. Male gender was strongly associated with vaccine acceptance, with an OR of 17.48 (95% CI: 8.58–35.63, *p* = 0.001) relative to females. In contrast, female gender was associated with vaccine reluctance or uncertainty, with an OR of 15.08 (95% CI: 6.65–34.18, *p* = 0.001). Parents who believed that returning to school was safe showed increased vaccine acceptance, with an OR of 4.32 (95% CI: 1.38–13.48, *p* = 0.01) compared to those who considered it not safe. Having children aged 12–18 years or aged 5–18 years showed no significant associations with reluctance (OR = 1.11, 95% CI [0.40–3.08], *p* = 0.84; and OR = 0.83, 95% CI [0.40–1.72], *p* = 0.62, respectively). No significant associations were observed for most education levels; Table 2 provides further details.

### 3.5. Study of Parental Perceptions Toward the Safety and Efficacy of COVID-19 Vaccine

**Figure 2:**
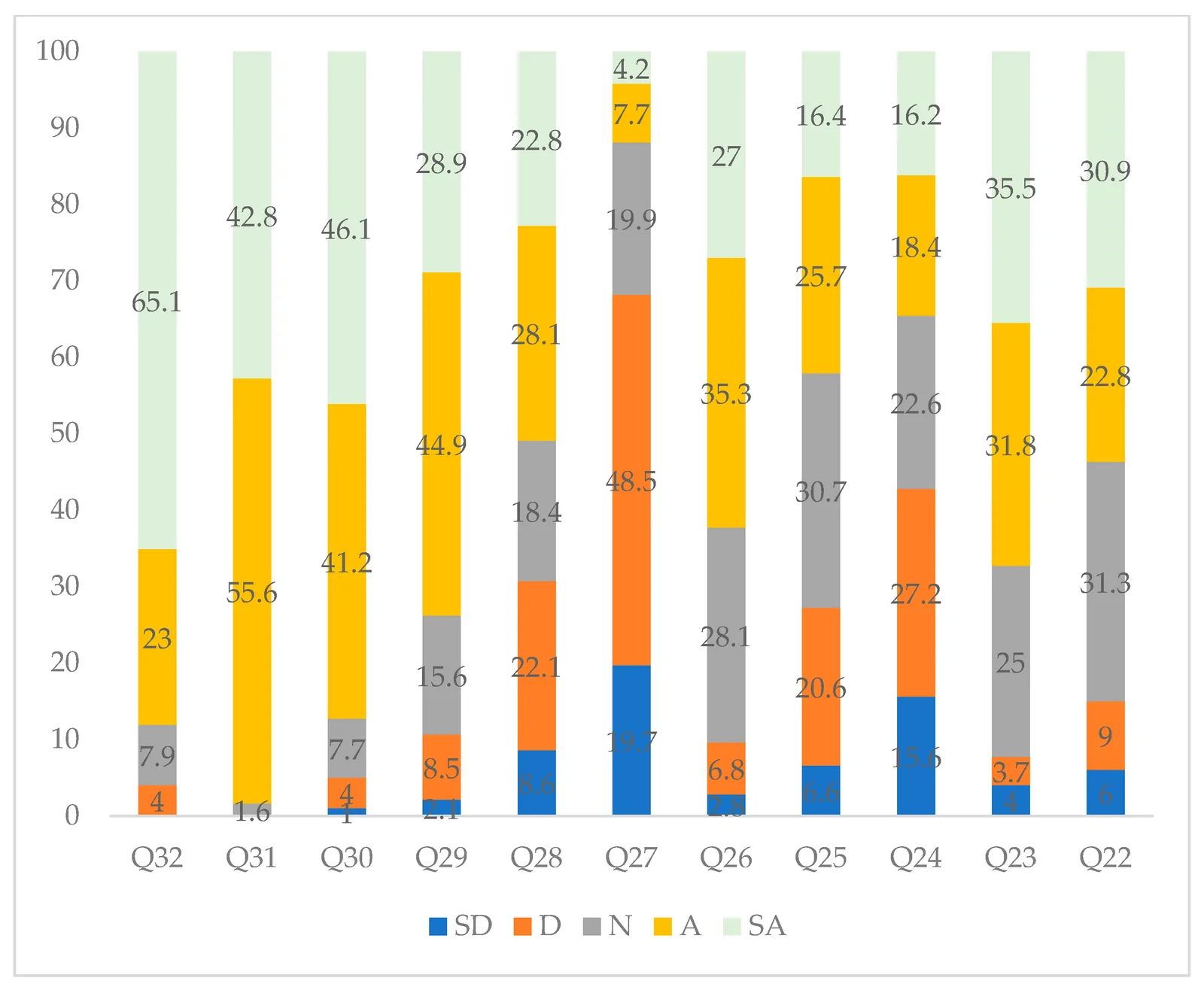
Percentage of parental agreement for the five psychological antecedents.

Each of the five psychological antecedents was assessed using a five-point Likert agreement scale. For analysis, respondents who selected a “neutral” response were classified as uncertain regarding that antecedent. Those who selected “strongly agree” or “agree” were categorized as accepting or influenced by the antecedent, whereas those who selected “strongly disagree” or “disagree” were classified as rejecting or unaffected by the antecedent. Statistically significant differences across the three parent groups (children aged 5–18 years, 5–11 years only, and 12–18 years only) were observed for multiple items, including confidence in vaccine safety and efficacy (Q22, *p* = 0.016), trust in Saudi health authorities (Q23, *p* = 0.015), beliefs about children’s immune system strength (Q25, *p* < 0.001), practical constraints such as transportation stress (Q27, *p* < 0.001), influence of media reports on vaccine hesitancy (Q28, *p* = 0.025), benefit–danger weighing (Q29, *p* = 0.008), need for vaccination understanding (Q30, *p* = 0.007), and collective responsibility items (Q31 and Q32, both *p* < 0.001). No significant difference was found regarding the perceived necessity of childhood vaccination to stop prior pandemics (Q24, *p* = 0.19) or the belief that childhood vaccines improve immune system effectiveness (Q26, *p* = 0.27). Figure 2 presents the percentage of parental agreement for each of the five psychological antecedents.

### 3.6. Multivariate Analysis of Relations Between the Psychological Factors

**Figure 3:**
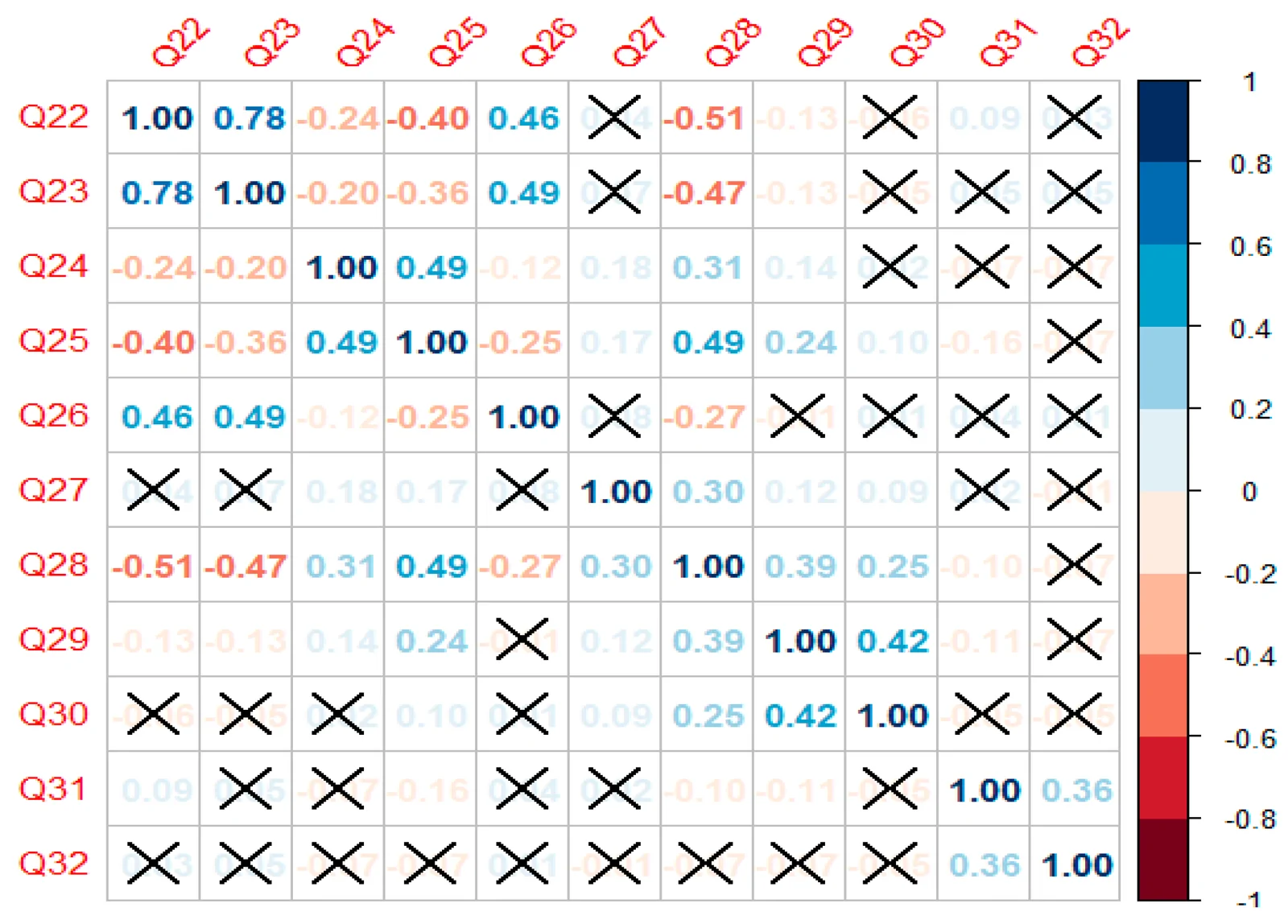
Correlation matrix of the 5C psychological determinants (Q22–Q32). Cells display Pearson correlation coefficients (range: −1 to +1). Positive correlations are shown in blue, negative correlations in red, with color intensity indicating the strength of the association (darker = stronger). Variable labels (Q22–Q32) correspond to the 5C questionnaire items (confidence, complacency, constraints, calculation, and collective responsibility).

Figure 3 presents the correlation matrix of responses to questions Q22 through Q32, which assess the 5C psychological determinants. The matrix displays Pearson correlation coefficients (r), where values approaching +1 or −1 indicate strong positive or negative correlations, respectively. Positive correlations are depicted in blue and negative correlations in red, with color intensity and circle size proportional to the coefficient magnitude, while all non-significant correlations (*p* > 0.05) are marked with an “X.” From the multivariable model, the following significant correlations were observed. Confidence in vaccine safety and efficacy (Q22) was strongly positively correlated with trust in Saudi health authorities (Q23) (r = 0.78). Belief that childhood vaccines improve immune system effectiveness (Q26) showed a moderate positive correlation with confidence in authorities (Q23) (r = 0.49). Hesitancy due to media reports about unpredictable adverse effects (Q28) was positively correlated with the belief that childhood vaccination is unnecessary to stop prior pandemics (Q24) (r = 0.31). The belief that children’s immune systems can protect them from COVID-19 (Q25) showed a moderate positive correlation with media-driven hesitancy (Q28) (r = 0.49). Collective responsibility to overcome the pandemic through universal immunization (Q31) was positively correlated with collective responsibility to protect vulnerable persons (Q32) (r = 0.36). Finally, negative correlations were observed between confidence in vaccine safety (Q22) and complacency-related beliefs, including the perceived unnecessary nature of childhood vaccination (Q24, r = −0.24) and the strength of children’s natural immunity (Q25, r = −0.40).

### 3.7. Study of the Impact of 5C Psychological Factors on Parental Decisions and Intentions to Vaccinate Children

Multiple logistic regression findings, presented in Table 3, revealed variable correlations between the 5C psychological antecedents and parental intention across different pediatric age groups (5–11 years, 12–18 years, and 5–18 years). Level of confidence was positively associated with parental intention to vaccinate children against COVID-19, with odds ratio estimates of 1.39 (*p* = 0.03) for the 5–11 years group, 1.53 (*p* = 0.05) for the 12–18 years group, and 1.23 (*p* = 0.01) for the 5–18 years group. Collective responsibility demonstrated similar positive associations with parental vaccination intentions across all age groups, with OR estimates of 2.13 (*p* = 0.05) for children aged 5–11 years, 1.33 (*p* = 0.03) for children aged 12–18 years, and 1.69 (*p* = 0.01) for children aged 5–18 years. Complacency was negatively associated with reluctance (i.e., uncertainty or rejection of the COVID-19 vaccine) among children aged 5–11 years (OR = 0.90, *p* = 0.01) and positively associated with reluctance among children aged 5–18 years (OR = 1.44, *p* = 0.05); no significant association was observed for the 12–18 years age group. Constraints demonstrated a negative association with parental intention to vaccinate children exclusively in the 5–11 years age group (OR = 0.58, *p* = 0.04). This construct exhibited the smallest overall effect on parental intention among all five psychological antecedents. Calculation was significantly associated with both intention and hesitation across children of varying ages: for the 5–11 years age group, the OR for acceptance was 0.09 (*p* = 0.03), and for the 5–18 years age group, the OR for uncertainty or reluctance was 1.11 (*p* = 0.05). Levels of confidence and collective responsibility were substantially correlated with parental intention to vaccinate children against COVID-19, particularly in the 5–11 years and 5–18 years age groups. As children grew older, parents became more receptive to COVID-19 vaccination. The presence of a child aged 5–11 years appeared to exert a unique influence on parental decision-making, affecting either enthusiastic approval or outright rejection of the vaccine. This phenomenon underscores the complex interrelationships between parents, children, and public health. Lower levels of constraints and calculation were associated with acceptance of the COVID-19 vaccine (*p* = 0.001). Positive correlations were also observed between confidence and collective responsibility and the willingness to receive the COVID-19 vaccine (*p* = 0.001).

## 4. Discussion

This study provides one of the first assessments of parental COVID-19 vaccination decisions for children in Saudi Arabia using the validated 5C psychological antecedents framework. Among 2176 parents, we observed a striking discordance: while over three-quarters of parents (77.9%) accepted vaccination for themselves, willingness to vaccinate their children declined sharply with younger age—58.6% for children aged 12–18 years, but only 41.9% for those under 12 years. Fathers were significantly more likely to accept vaccination than mothers (OR = 17.48), and having a child aged 5–11 years increased parental reluctance threefold (OR = 3.16). Confidence and collective responsibility emerged as the strongest positive predictors of vaccine acceptance, particularly for younger children (OR up to 2.13), whereas extensive information-seeking (calculation) was associated with lower acceptance. Taken together, these findings indicate that parental hesitancy in Saudi Arabia is not uniform; it varies markedly with child age and is driven less by practical barriers than by trust, social responsibility, and information processing.

The finding that parental acceptance drops sharply for children under 12 years aligns with international reports. Kitro et al. found that only 32% of Thai parents were willing to vaccinate children aged 5–11 years [21]; in the United States, Ruggiero et al. and Teasdale et al. reported similar age-dependent declines [22,23], and Zychlinsky Scharff et al. identified that students’ age and parental education level influence COVID-19 vaccination hesitancy [24]. This consistent pattern suggests that younger child age functions as a universal psychological barrier, likely because parents perceive younger children as more vulnerable to vaccine side effects and less at risk from severe COVID-19. Notably, in our study, parents of children aged 5–11 years showed the highest proportion of “Yes” responses (20.2%) when asked specifically about the newly announced pediatric vaccine, indicating that once a vaccine is officially authorized for younger children, a substantial minority remain willing—but the majority still hesitate. This gap between adult self-vaccination (77.9%) and child vaccination (41.9%)—an approximately 36 percentage point difference—represents a critical target for public health intervention.

A notable gender difference emerged. Fathers were significantly more likely to accept COVID-19 vaccination for their children than mothers (OR = 17.48), and mothers correspondingly showed higher reluctance (OR = 15.08). This disparity is consistent with Horiuchi et al.’s findings in Japan and Troiano and Nardi’s international analysis [25,26], though Fedele et al. did not observe a gender gap in an Italian population [27]. The difference may stem from differential risk perception and information-seeking behavior. Mothers often bear primary responsibility for children’s health decisions and may engage in more extensive information seeking, which—when faced with contradictory online content—can paradoxically increase uncertainty. Fathers may place greater trust in health authority recommendations and view vaccination as a straightforward disease prevention measure. In Saudi Arabia’s family structure, where mothers frequently manage day-to-day child health, public health messaging should specifically address mothers’ concerns through trusted channels such as pediatric well-child visits and school-based communications.

A counterintuitive finding concerned COVID-19 hospitalization history. A history of COVID-19 hospitalization among family or friends was significantly associated with increased parental reluctance (*p* = 0.03). This contrasts with the assumption that severe illness exposure would boost vaccine acceptance. One possible explanation is that witnessing a severe COVID-19 case may heighten generalized health anxiety, which can manifest as fear of any medical intervention—including vaccines. Alternatively, parents who saw a relative hospitalized but recover might perceive COVID-19 as survivable without vaccination. Yigit et al. and Szilagyi et al. similarly reported that insufficient information about vaccine safety, rather than disease severity perception, was the primary driver of refusal [28,29]. This finding underscores the need for healthcare providers to address post-hospitalization emotional distress and provide clear, separate risk communications about vaccination.

One possible explanation for this counterintuitive finding is that witnessing severe COVID-19 illness in one’s social network may heighten anxiety and fear, which, in some cases, may paradoxically lead to avoidance of vaccination due to heightened perceptions of vaccine risks or distrust in pharmaceutical interventions, particularly in a context where misinformation about vaccines circulates widely on social media. Additionally, in KSA, where the majority of COVID-19 hospitalizations were managed in public health facilities, negative experiences with healthcare settings during such events may have eroded trust in the system, thereby contributing to vaccine reluctance. However, it is important to emphasize that this interpretation is speculative and hypothesis-generating, and further qualitative research is needed to explore this association in depth.

Perceptions of school safety also influenced decisions. Parents who believed that returning to school was safe showed significantly higher vaccine acceptance (OR = 4.32, *p* = 0.01). This is consistent with Zhang et al., who identified school return as a key decisional factor [30], and with Aljamaan et al., who explored in-person schooling amidst children’s COVID-19 vaccination during the Omicron variant announcement in Saudi Arabia [31]. In Saudi Arabia, where in-person schooling resumed during the study period, parents faced a practical trade-off: vaccinating children enabled normal school attendance, while hesitancy risked continued educational disruption. Accordingly, framing pediatric COVID-19 vaccination as an enabler of normal childhood activities—rather than solely as a disease prevention measure—may resonate with hesitant parents.

Our central contribution is the application of the 5C framework to pediatric COVID-19 vaccination in an Arab population. Confidence was positively associated with acceptance across all age groups (OR 1.23–1.53), and collective responsibility showed even stronger associations (OR 1.33–2.13), particularly for younger children. These findings align with Lazarus et al.’s global survey and Elbarazi et al.’s qualitative Arab study, both of which identified trust in authorities and desire to protect others as core drivers of vaccine acceptance [32,33]. Saudi Arabia’s high adult vaccination rate (92% completed second dose in our sample) likely reflects a cultural emphasis on collective obligation—a value that public health campaigns can explicitly leverage. Indeed, Abdou et al. found that collective responsibility was a key predictor across 13 Arab countries using the Arabic 5C tool [34], and Alenezi et al. confirmed that parental perceptions and the 5C antecedents during the Omicron surge in Saudi Arabia were strongly influenced by confidence and collective responsibility [35]. Messaging that emphasizes protecting vulnerable groups—for example, “vaccinating your child protects not only them but also grandparents, classmates with chronic illnesses, and the wider community”—directly activates collective responsibility.

Calculation (active information seeking) was associated with lower acceptance (OR = 0.09 for children aged 5–11 years, *p* = 0.03). This is a crucial finding: parents who searched extensively for information were more hesitant, not less. In the context of COVID-19, where social media platforms hosted rapidly evolving and often contradictory content, extensive searching may have exposed parents to unverified safety concerns, rare adverse event reports, and conspiracy theories. This does not mean that information should be withheld; rather, healthcare authorities must provide authoritative, easy-to-find, and consistently updated information that pre-emptively answers common questions. Ghazy et al. previously demonstrated that the Arabic 5C scale can identify high-calculation individuals who benefit from targeted, simplified communication [19]. Similar patterns of vaccine hesitancy influenced by information seeking have been observed internationally, including in the USA, Canada, Italy, and South Korea [36,37,38,39,40].

Complacency showed a mixed pattern: negatively associated with reluctance in the 5–11 years group (OR = 0.90, *p* = 0.01) but positively associated with reluctance in the 5–18 years group (OR = 1.33, *p* = 0.05). This suggests that among parents of younger children, low perceived disease risk (complacency) actually reduced reluctance—perhaps because they did not feel urgency. Conversely, among parents of older children, complacency increased reluctance, possibly because older children have higher baseline exposure risk. Constraints (practical barriers) had the smallest overall effect, with statistical significance only for the 5–11 years group (OR = 0.58, *p* = 0.04). This indicates that in Saudi Arabia, logistical issues such as transportation, appointment availability, and cost are not the primary obstacles—psychological factors dominate. This finding is consistent with Alsubaie et al. on vaccine hesitancy among Saudi parents [17] and Aldakhil et al. on childhood immunizations during COVID-19 [41].

The correlation matrix (Figure 3) reveals several important relationships. Confidence in vaccine safety (Q22) was strongly correlated with trust in Saudi health authorities (Q23, r = 0.78), indicating that institutional trust is the bedrock of vaccine confidence. Conversely, confidence was negatively correlated with complacent beliefs that children’s natural immunity is sufficient (Q25, r = −0.40) and that childhood vaccination is unnecessary (Q24, r = −0.24). This reinforces the idea that building confidence requires directly countering complacency narratives. The positive correlation between media-driven hesitancy (Q28) and belief that childhood vaccination is unnecessary (Q24, r = 0.31) suggests that exposure to social media debates may actively foster complacent attitudes. Alnasser et al. similarly highlighted how vaccine hesitancy among Saudi parents is shaped by these intersecting psychological factors [42].

Several limitations should be acknowledged. First, the snowball sampling method via social media may introduce selection bias, overrepresenting digitally literate parents and underrepresenting those without social media access. Second, all data are self-reported, and stated vaccination intention may not perfectly correlate with actual uptake—though intention is a strong predictor of behavior. Third, the sample was predominantly female (63.6%) and from the Qassim region (56.8%), which may limit generalizability to other Saudi regions or to male-only samples. Fourth, the cross-sectional design precludes causal inference. Fifth, parents who had already vaccinated their children (as required for eligibility) may have different attitudes than the general parent population—though this reflects real decision-makers. Finally, social desirability bias may have influenced responses, particularly regarding collective responsibility items.

Despite these limitations, several actionable recommendations emerge. To enhance confidence, public health campaigns should emphasize transparent safety data, secure endorsements from credible Saudi health authorities, and incorporate firsthand accounts from pediatricians. Collective responsibility messaging should be explicitly framed around protecting vulnerable groups. To address calculation-driven hesitancy, authorities should create a single, easy-to-navigate digital portal (such as a Ministry of Health website with an FAQ section specifically for parents of 5–11 year olds) that directly answers common safety questions, rather than forcing parents to search across social media. School-based vaccination campaigns and parent education sessions can leverage the finding that school-return safety beliefs strongly predict acceptance. Targeted interventions should be developed for mothers, who show higher reluctance, perhaps through pediatric well-child visits or female-focused social media influencers. Finally, the finding that constraints (logistical barriers) have minimal influence suggests that resources are better spent on psychological interventions than on reducing practical barriers, at least in the Saudi context. In comparison with earlier studies conducted in Saudi Arabia, such as those by Alenezi et al. (2022) [35] and Abdou et al., [34] our study offers a novel contribution through its age-stratified analytical approach. While previous work identified general 5C predictors of hesitancy, we show that these predictors operate differently depending on the child’s age, with confidence and collective responsibility being particularly critical for parents of children aged 5–11 years. This age-specific insight has not been previously documented and carries important implications for designing targeted public health interventions.

## 5. Contextualizing Findings Within Subsequent Developments

Given the time elapsed since data collection, we acknowledge the need to contextualize our findings within subsequent developments. Several significant developments have occurred since our data collection period that contextualize our findings. In Saudi Arabia, COVID-19 vaccination policy for children evolved rapidly: Pfizer-BioNTech was approved for children aged 12–18 years in June 2021, followed by approval for those aged 5–11 years in November 2021, coinciding with the start of our survey [43]. Subsequently, the Saudi Ministry of Health launched a dedicated pediatric vaccination campaign (“It is our turn now”) to encourage uptake among children aged 5–11 years, achieving a 60% willingness rate among parents to vaccinate their children [44]. More recently, a 2025 systematic review and meta-analysis from Saudi Arabia reported a pooled parental willingness rate of 48.0% (95% CI: 41.0–54.0%) for childhood COVID-19 vaccination, with perceived vaccine safety and efficacy emerging as the most significant determinants [45]. This rate is slightly higher than the 41.9% acceptance we observed for children younger than 12 years, suggesting a modest upward trend over time, though substantial hesitancy persists. Despite these temporal changes, our psychological findings remain highly relevant for future pandemic preparedness in Saudi Arabia and beyond. The 5C antecedents—particularly confidence and collective responsibility—were the strongest positive predictors of vaccine acceptance, a pattern corroborated in subsequent research conducted during the Omicron surge [35]. Our identification of calculation (extensive information seeking) as a barrier to acceptance also aligns with contemporary evidence that negative situational cues—including anecdotal experiences and social media content—significantly influence novel vaccine decisions [35]. Therefore, while the specific policy context has evolved, the core psychological drivers we identified are enduring and should inform the design of tailored communication strategies in any future pandemic scenario.

## 6. Conclusions

Parental attitudes toward childhood COVID-19 vaccination are crucial for herd immunity. This study identifies key influencing factors: child age, parental gender, prior COVID-19 hospitalization, school safety perceptions, and the 5C psychological antecedents. Confidence and collective responsibility were the strongest positive predictors of acceptance, particularly for children aged 5–11 years, while practical constraints had the smallest effect. Few participants worried about side effects or logistical issues. However, it is important to interpret these findings with caution, as our sample was restricted to parents who had already initiated vaccination for their children; thus, our reported acceptance rates may overestimate population-level willingness. Nevertheless, by addressing parental concerns and providing clear safety information, healthcare providers can increase vaccination rates. Building public confidence and fostering collective responsibility should remain central priorities for public health campaigns in Saudi Arabia. However, these conclusions are most applicable to parents who have already initiated vaccination, and further research is needed to understand the drivers of complete refusal among the broader parent population.

## Figures and Tables

**Table 1:** Overall characteristics of participants stratified by the age range of their children.

Variables	Parents of Children Aged 5–18 yrs. n = 568 (26.1%)	Parents of Children Aged 5–11 yrs. n = 1276 (58.64%)	Parents of Children Aged 12–18 yrs. n = 332 (15.26%)	Total 2176 (100%)	*p*-Value
Gender					0.01 *
Male	180 (8.27%)	428 (19.67%)	184(8.46%)	792 (36.4%)	
Female	388 (17.83%)	848 (38.97%)	148 (6.8%)	1384 (63.6%)	
Nationality					0.93
Saudi	540 (24.82%)	1212 (55.7%)	312 (14.34%)	2064 (94.85%)	
Non-Saudi	28 (1.29%)	64 (2.94%)	20 (0.92%)	112 (5.15%)	
Region					0.06
Riyadh	112 (19.7%)	280 (21.9%)	72 (21.7%)	464 (21.3%)	
Makkah	16 (2.8%)	52 (4.1%)	12 (3.6%)	80 (3.7%)	
Madinah	0 (0.0%)	4 (0.3%)	0 (0.0%)	4 (0.2%)	
Qassim	300 (52.8%)	744 (58.3%)	192 (57.8%)	1236 (56.8%)	
Eastern	56 (9.9%)	112 (8.8%)	24 (7.2%)	192 (8.8%)	
Asir	4 (0.7%)	24 (1.9%)	8 (2.4%)	36 (1.7%)	
Tabuk	16 (2.8%)	16 (1.3%)	0 (0.0%)	32 (1.5%)	
Hail	12 (2.1%)	4 (0.3%)	4 (1.2%)	20 (0.9%)	
The Northern Border	0 (0.0%)	4 (0.3%)	0 (0.0%)	4 (0.2%)	
Jazan	4 (0.7%)	4 (0.3%)	0 (0.0%)	8 (0.4%)	
Najran	4 (0.7%)	0 (0.0%)	4 (1.2%)	8 (0.4%)	
Al Baha	28 (4.9%)	8 (0.6%)	8 (2.4%)	44 (2.0%)	
AL Jouf	8 (1.4%)	8 (0.6%)	4 (1.2%)	20 (0.9%)	
Out of KSA	8 (1.4%)	16 (1.3%)	4 (1.2%)	28 (1.3%)	
Receive 2nd dose of COVID-19					0.07
Yes	559 (25.69%)	1186 (92.95%)	256 (11.76%)	2001 (91.96%)	
No	9 (0.41%)	80 (3.68%)	86 (3.95%)	175 (8.00%)	
Education					0.05 *
Student	160 (28.2%)	36 (2.8%)	4 (1.2%)	200 (9.2%)	
Technician Diploma	68 (12.0%)	112 (8.8%)	40 (12.0%)	220 (10.1%)	
Bachelor Degree	268 (47.2%)	816 (63.9%)	188 (56.6%)	1272 (58.5%)	
Higher Diploma	4 (0.7%)	68 (5.3%)	16 (4.8%)	88 (4.0%)	
Postgraduate Studies	32 (5.6%)	144 (11.3%)	36 (10.8%)	212 (9.7%)	
Illiterate	4 (0.7%)	0 (0.0%)	0 (0.0%)	4 (0.2%)	
Others	32 (5.6%)	100 (7.8%)	48 (14.5%)	180 (8.3%)	
Health Worker					0.21
Yes	36 (6.3%)	88 (6.9%)	16 (4.8%)	140 (6.4%)	
No	532 (93.7%)	1188 (93.1%)	316 (95.2%)	2036 (93.6%)	

* *p* < 0.05 (significant).

**Table 2:** Multiple logistic regression assessing parental intention to vaccinate children based on children’s age group.

Variables	YES (Acceptants)			No/Unsure (Reluctants)		
	OR	95%CI	*p*-Value	OR	95%CI	*p*-Value
Children age group						
Having children (5–11)	1.00	[0.54–1.86]	0.99	3.16	[1.57–6.37]	0.001 *
Having children 12–18	0.71	[0.33–1.55]	0.39	1.11	[0.40–3.08]	0.84
Having children 5–18	0.24	[0.14–0.42]	0.05 *	0.83	[0.40–1.72]	0.62
Gender of parents						
Male	17.48	[8.58–35.63]	0.001 *	0.96	[0.46–1.77]	0.90
Female	2.38	[0.67–8.44]	0.18	15.08	[6.65–34.18]	0.001 *
Parental level of Education						
Student	0.53	[0.23–1.04]	0.19	0.26	[0.03–2.14]	0.18
Technician Diploma	1.00	[0.18–1.40]	0.54	1.23	[0.24–8.95]	0.82
Bachelor Degree	0.09	[0.04–0.21]	0.05 *	0.17	[0.01–3.71]	0.89
Higher Diploma	1.23	[0.23–2.12]	0.89	1.46	[0.27–2.65]	0.26
Postgraduate Studies	0.77	[0.29–2.03]	0.39	0.54	[0.24–1.24]	0.43
Illiterate	0.24	[0.03–1.88]	0.08	0.11	[0.02–0.57]	0.009 *
Others	1.00	[0.94–1.06]	0.09	0.84	[0.42–1.67]	0.09
Return to school						
Safe	4.32	[1.38–13.48]	0.01 *	0.29	[0.14–0.59]	0.01 *
Not safe	0.34	[0.12–0.95]	0.05 *	0.31	[0.04–1.94]	0.07

OR: Odds Ratio; * Significant.

**Table 3:** Multiple logistic regression assessing the impact of 5C psychological antecedents.

Variables	YES (Acceptants)			No/Unsure (Reluctants)		
	OR	95%CI	*p*-Value	OR	95%CI	*p*-Value
Confidence						
Having children 5–11	1.39	[1.27–1.52]	0.03 *	0.91	[0.13–6.37]	0.92
Having children 12–18	1.53	[1.37–2.65]	0.05 *	1.11	[0.40–3.08]	0.84
Having children 5–18	1.23	[0.33–1.32]	0.56	0.87	[0.33–1.54]	0.70
Complacency						
Having children 5–11	0.90	[0.46–1.77]	0.76	15.08	[8.58–26.49]	0.01 *
Having children 12–18	2.38	[0.67–8.44]	0.99	11.81	[10.65–13.09]	0.01 *
Having children 5–18	1.44	[1.33–1.55]	0.05*	13.81	[10.65–17.18]	0.01 *
Constraints						
Having children 5–11	0.58	[0.24–1.38]	0.22	0.29	[0.08–1.05]	0.06
Having children 12–18	0.78	[0.18–3.38]	0.86	0.09	[0.02–0.39]	0.001 *
Having children 5–18	1.27	[0.32–1.35]	0.42	0.83	[0.18–3.68]	0.06
Calculations						
Having children 5–11	0.09	[0.02–0.39]	0.03 *	1.23	[0.21–7.21]	0.82
Having children 12–18	0.83	[0.12–1.35]	0.42	1.17	[0.33–1.55]	0.16
Having children 5–18	1.00	[0.34–1.94]	0.06	1.11	[1.09–1.87]	0.05 *
Collective responsibility						
Having children 5–11	2.13	[1.79–2.47]	0.01 *	0.78	[0.45–1.35]	0.73
Having children 12–18	1.33	[0.98–1.77]	0.05 *	2.80	[0.79–9.92]	0.09
Having children 5–18	1.69	[1.51–1.88]	0.01 *	1.62	[0.33–7.96]	0.17

OR: Odds Ratio; * Significant.

## Data Availability

This publication contains all of the information analyzed during this investigation.
